# Secretome of Mesenchymal Stromal Cells as a Possible Innovative Therapeutic Tool in Facial Nerve Injury Treatment

**DOI:** 10.1155/2023/8427200

**Published:** 2023-01-14

**Authors:** Ilona Szabłowska-Gadomska, Stefan Rudziński, Marta Dymowska

**Affiliations:** ^1^Laboratory for Cell Research and Application, Center for Preclinical Research and Technology, Medical University of Warsaw, Banacha 1b, 02-097 Warsaw, Poland; ^2^BBMRI.pl Consortium, Warsaw, Poland

## Abstract

Facial nerve palsy is a serious neurological condition that strongly affects patient everyday life. Standard treatments provide insufficient improvement and are burdened with the risk of severe complications, e.g., facial synkinesis. Mesenchymal stromal cell-based therapies are a novel and extensively developed field which offers new treatment approaches with promising results in regards to the nervous tissue regeneration. The potential of mesenchymal stromal cells (MSCs) to aid the regeneration of damaged nerves has been demonstrated in several preclinical models, as well as in several clinical trials. However, therapies based on cell transplantation are difficult to standardize in the manner similar to that of routine clinical practices. On the other hand, treatments based on mesenchymal stromal cell secretome harness the proregenerative features of mesenchymal stromal cells but relay on a product with measurable parameters that can be put through standardization procedures and deliver a fully controllable end-product. Utilization of mesenchymal stromal cell secretome allows the controlled dosage and standardization of the components to maximize the therapeutic potential and ensure safety of the end-product.

## 1. Introduction

Facial nerve paralysis (FNP) is a major neurological condition that causes aesthetic and psychological distress as well as motor dysfunction, degrading the quality of patient life and eventually leading to social exclusion of most sensitive patients [1]. Facial nerve (FN) is one of the major cranial nerves that descends directly from the brain and relay information to and from head and neck. FN controls many important functions, i.e., facial muscle movement or parasympathetic regulation of submandibular and maxillary salivary glands and lacrimal glands. It also conveys the information from receptors located in the frontal part of tongue and both soft and hard palates, including the taste receptors. Moreover, facial nerve controls orbicularis oculi muscle that partakes in the eyelid closing process. For this reason, the FN dysfunction may also result in sight weakening due to continuous irritation of cornea by external factors [2]. FNP occurs in 20-30 cases per 100,000 people [3]. There are numerous potential causes of FNP, among which the most frequent are viral infection, e.g., herpes simplex virus, shingles (varicella zoster virus infection), skull injuries, inflammatory diseases of the middle ear, metabolic diseases, and tumors. However, finding the direct cause of FNP is often difficult; therefore, 60-70% of FNP cases are diagnosed as an idiopathic facial nerve palsy or Bell's palsy [3]. In Poland, about 8 thousand people are affected by FNP each year, and 5% of those cases turn out to be chronic palsy [4].

## 2. Current Treatment Approach

Standard treatment approach based on pharmacotherapy and physiotherapy proves effective only for the patients who were diagnosed in the early stage of FNP. For those in severe medical condition, often associated with some complications, e.g., patients with nerve rupture or chronic idiopathic palsy symptoms, standard treatment is ineffective. For this group of patients, the most commonly used treatment is surgical intervention. The facial nerve surgery aims to restore facial symmetry and, at least partially, the ability to move facial muscles. In cases of nerve disruption, surgical connection of ruptured nerve is a highly effective treatment. However, if the nerve disruption is larger due to the mechanical damage or tumor related nerve damage, the connective surgery is not possible. In these cases, nerve transplantation or nerve substitution is required [5, 6]. Unfortunately, these methods pose a high risk of complications. The success rate of the aforementioned surgical procedures is determined by several factors such as a time between injury occurrence and surgery, precise adjustment of the transplanted nerve size, and lack of tension at the connection site [6].

In nerve transplantation procedure of the facial nerve, most commonly used nerve donors are the sural nerve, located in the back of the calf, which provides the sensation to the lower lateral leg, or the great auricular nerve, providing the sensation to the earlobe. They are chosen for their sufficient length and structural similarity to the facial nerve. However, surgical intervention in nerve injury treatment is associated with high risk of complications, such as facial synkinesis—spontaneous, involuntary contractions of facial muscles which interfere in facial expression and physiological function [7] and pain accompanying jaw movements. There is also a risk of causing the donor nerve impairment, while resecting the tissue for surgery. Another surgical method that restores functionality and shape of patient face is the hypoglossal-facial nerve anastomosis. This intervention uses a hypoglossal nerve, which innervates the muscles of the tongue, as a donor motor nerve for reinnervation of facial muscles. However, it comes with a potential risk of difficulties with pronunciation or loss of sensation in the tongue [8].

In summary, the standard treatment of chronic facial nerve dysfunctions is, at present, the surgical reconstruction, e.g., autologous nerve transplantation or nerve anastomosis. However, the nerve transplantation procedures have their limitations, mostly due to the loss of neurological functions at the site of nerve harvesting, which decreases the number of places from which the transplant sample can be acquired [9].

Despite intensive research towards finding new surgical techniques of FNP treatment, the current methods do not enable the restoration of full nerve functionality. Numerous preclinical studies and clinical trials have been conducted in the field of facial nerve reconstruction surgery. Unfortunately, no spectacular success has been noted over the span of the last several years. The lack of progress and the burden of complications related to currently used methods shift the weight of research projects towards searching for novel therapeutic approaches, including those offered by regenerative medicine.

## 3. Cell Treatment–Innovative Approach in FNP Treatment

Regenerative medicine is an interdisciplinary field that focuses on development of new therapies that will allow for replacement, repair, and regeneration of diseased or damaged tissue and organs [10, 11]. Cell-based therapies constitute important and dynamically developing arm of regenerative medicine, and the special attention is devoted to stem-cell-based approaches. Stem cells differ from other cells in their ability to self-renew and potential to differentiate into various cell types. These unique properties allow them to maintain tissue homeostasis under physiological conditions as well as to play a role in regeneration of damage caused by trauma or disease.

Numerous studies are underway to develop methods based on stem cells; however, their application in routine therapies is still the future. This is mainly due to the lack of understanding of the actual therapeutic mechanisms of these cells after their application to the patient and the lack of unequivocal evidence confirming the safety of this method in various indications. Therefore, it is highly important to conduct research on stem cells, towards the development of effective, efficient, inexpensive, but most of all, safe methods of stem cell acquisition, isolation, *in vitro* culture, and preparation of the stem-cell-based therapeutics [12]. According to http://Clinicaltrial.gov, the website curated by U.S. National Institutes of Health, there are over 10,000 active clinical trials utilizing stem cells [13].

Among different types of stem cells, MSCs (also called the mesenchymal stromal cells) deserve special attention. According to The International Society for Cellular Therapy (ISCT), there are a few basic criteria for classification of these morphologically close to fibroblasts MSCs: the ability to adhere to the culture vessel *in vitro* as well as the presence of CD73, CD90, and CD105 surface antigens and the lack of CD34, CD45, CD14 or CD11b, and CD79*α* or CD19 antigens, and MHC class II markers. Next, vital criterion is their ability to differentiate into osteoblasts, adipocytes, and chondroblasts [14]. It has been proven that MSCs can also differentiate into cells other than those of mesodermal germ layer, e.g., into hepatocytes or neurons [15, 16]. Moreover, anti-inflammatory, antiapoptotic, and immunomodulatory properties were attributed to MSCs [17–19]. Mesenchymal stromal cells affect the functions of many types of immune cells, i.e., they inhibit T lymphocyte proliferation, cytotoxicity and cytokine secretion [20, 21], proliferation of B lymphocytes, and antibody production [22], and they also suppress IL-2-induced proliferation of resting natural killer cells [23], inhibit maturation and activation of dendritic cells [24, 25], and induce the switch in macrophages from pro-inflammatory M1 to anti-inflammatory M2 phenotype [26].

MSCs can be isolated from various sources, i.e., bone marrow, Wharton's jelly, umbilical cord blood, or adipose tissue. Formerly, MSCs were isolated mainly from the bone marrow; however, adipose tissue is recently regarded as a more attractive source of MSCs due to high accessibility. Adipose tissue is the niche for adipose-derived stromal cells (ADSCs) that can be obtained during the liposuction, a cosmetic procedure of fat removal from the specific area of the body. The invasiveness of liposuction procedure is relatively low, and material acquired following this procedure, usually regarded as a medical waste, is an abundant source of mesenchymal stromal cells. Research on dogs showed that adipose tissue can yield even 500-fold more MSCs than the bone marrow in which MSC fraction constitutes merely 0.001-0.01% of all cells [27]. In humans, this ratio may reach a similar value [28]. This abundance of MSCs in lipoaspirates allows researchers to preserve cell populations which have been already well characterized and proven useful for the future use. There are also biobanks spread all over the world, storing mesenchymal stromal cells for the research use and providing appropriately cryopreserved and well characterized cells.

Due to their unique properties, MSCs are a subject to numerous studies and clinical trials in various fields of medicine. There are over 7,000 clinical trials ongoing worldwide in which MSCs are involved [13]. MSCs are most frequently used for the experimental treatment of cardiovascular diseases (15%), neurodegenerative diseases (12%), bone and cartilage disorders (6%), cancer (5%), liver diseases (3%), kidney diseases (3%), and autoimmune diseases (25%) including GvHD (graft-versus-host diseases), Crohn's disease, or type 1 diabetes [29].

Regarding the usefulness of MSCs in nerve regeneration, Lopatina et al. [30] have confirmed the beneficial effect of engrafted ADSCs in regeneration of peripheral nerve and nerve protrusion in the murine model. Furthermore, the authors showed that murine and human ADSCs produce neurotrophic factors and elements of myelin sheath that are vital for nerve growth and myelination. Induction of neural differentiation in ADSCs increases the production of brain-derived neurotrophic factor (BDNF) as well as capability of these cells to induce nervous fibers growth [30]. Promising results have also been obtained in experiments in rats showing that adipose-derived MSCs can differentiate into Schwann-like cells [31, 32]—the glial cells regulating regeneration of the injured nervous tissue. Moreover, Wang et al. [33] proved that introducing bone marrow-derived mesenchymal stromal cells (BMSCs) and ADSCs differentiated into Schwann-like cells to the acellular nerve allograft improved sciatic nerve regeneration. Interestingly, it was also demonstrated in a rat model that BMSCs expanded under hypoxic conditions promote nerve regeneration more effectively compared to BMSCs cultured in normoxia [34]. There are also reports indicating that MSCs can be directly used to treat nerve injuries in humans. Aggarwal et al. [35] demonstrated that injection of the BMSC concentrate into the site of nerve transection in patients with facial nerve paralysis resulted in the improvement in eye closure and facial deviation in comparison with conventional surgical treatment. However, the mechanism of action of MSCs in this case has not been described.

MSCs were originally thought to act via replacement mechanism in which they would migrate to the injured site and differentiate to replace damaged cells and tissues. It has also been demonstrated that MSCs can influence other types of cells through the cellular content exchange, including organelles like mitochondria. As an example, Jackson et al. [36] revealed that human BMSCs promote phagocytic capacity of macrophages through the mitochondrial transfer via tunneling nanotubes. However, growing body of evidence shows that direct cell-to-cell contact is not required for MSCs to achieve regenerative or immunomodulatory effect. Moreover, MSCs display low survival rates after systemic application, which in animal models can drop below 1% of original viability. It indicates that MSCs' therapeutic effect results from the activity of the substances secreted by these cells rather than their direct engagement in the reconstruction of damaged tissues or organs [37, 38]. The results obtained by Salomone et al. [39] may serve as an indirect confirmation of this theory. They demonstrated that in the rat model of neurotmesis of the mandibular branch of the facial nerve both undifferentiated BMSCs and Schwann-like cells differentiated from BMSCs improved facial nerve regeneration. However, the functional results were better when undifferentiated cells were used [39].

The broad spectrum of active factors secreted by MSCs draws growing attention, since they are very likely to be the key elements regulating the processes of regeneration and repair of damaged tissues and organs.

## 4. Secretome of MSCs

The MSC secretome is an array of water soluble factors produced by MSCs to communicate with other cells [40, 41]. Secretome consists of lipid transmitters (e.g., prostaglandin E2 and PGE2), genetic material (mRNA, miRNA), and soluble proteins, such as serum proteins, growth factors (transforming growth factor *β*, TGF-*β*; hepatocyte growth factor, HGF), hormones (insulin growth factor-1, IGF-1), cytokines (interleukin- (IL-) 6, IL-8, and IL-10), enzymes (indoleamine-2,3-deoxygenase, IDO; matrix metalloproteinases, MMPs), and extracellular matrix (ECM) proteins [41, 42]. There are two mechanisms of secretion of these molecules by MSCs: classical (endoplasmic reticulum- (ER-) Golgi pathway) and nonclassical, including extracellular vesicles (EVs) [41, 43–45].

EVs can be separated from soluble factors, and each fraction of secretome can be an independent product that will initiate various therapeutic mechanisms at the site of tissue or organ damage.

The term EV includes particles naturally secreted by cells that are limited by the lipid bilayer and are unable to replicate (e.g., do not have a functional nucleus) [46]. Inside the lipid bilayer, EVs can carry a various “cargo” originating from cellular cytoplasm, e.g., DNA fragments, mRNA, miRNA, or signaling particles. There are two pathways of the EV formation process. The first one is the ceramide-dependent pathway in which the lipid rafts are formed. They are subsequently enriched with ceramide which is a product of sphingomyelin conversion by sphingomyelinase. As ceramides are highly hydrophobic and have cone-shaped structure, their presence in the membrane causes spontaneous negative curvature that promotes exosome formation [47]. The second mechanism of EV formation is the ESCRT-dependent pathway (endosomal sorting complexes required for transport) that induces the migration of proteins and “cargo” to the membranes of endosomal bodies, e.g., multivesicular bodies (MVBs). Then, the membrane begins to invaginate until it closes up, forming a vesicle. After that, the components of ESCRT facilitate vesicle transport, first to the cytoplasm and then through the cell membrane into the ECM [48].

The application of MSC secretome allows elimination of numerous potential threats related to cell-based therapy without losing its therapeutic value [49]. First of all, secretome-based therapies are far safer than cell-based therapies as they pose lower risk of cancerogenesis, unwanted immune response, or virus transmission [50]. Products based on MSC secretome would be easier to assess qualitatively in terms of dose and potency, i.e., standard parameters assessed for conventional pharmaceutical products. Furthermore, the storage of secretome is easy and inexpensive compared to that of cells which are routinely stored in liquid nitrogen and require addition of potentially toxic cryoprotectants to sustain cell viability [51]. Since secretome-based therapies are cell-free, there is a lower likelihood of product delivery failure. The manufacturing process in which the end-products consist of fully grown and viable cell cultures is expensive, multistage, and time- and work-consuming. Moreover, it requires multistep preparations, coordination, precise planning, and synchronization with patients' condition due to the short expiration time of such products (less than 24 hours). The way to extend the product suitability to be applied to a patient is to freeze cells and then thaw them right before the application, even next to the patient's bed. However, products prepared in this way contain cryoprotectants which pose an additional threat for patient health. Moreover, freshly thawed cells have worse quality parameters (e.g., viability) than cells collected directly from a cell culture [52], which could have a serious impact on the therapeutic efficacy of the product. On the other hand, properly prepared MSC secretome could be an off-the-shelf type of product, meaning it could be applied directly after thawing at any time convenient for the patient.

Numerous clinical trials employing MSC secretome demonstrate its potential to aid the regeneration of damaged organs and tissues, including heart, kidney, muscle, and central nervous system (CNS) [53]. Zhou et al. [54] revealed that application of conditioned media (CM) harvested from adipose-derived stem cell culture improves wound healing after fractional CO2 laser resurfacing used in aesthetic medicine in order to reduce the appearance of wrinkles, scars, and skin discolorations. The positive effect of MSC-CM has also been demonstrated in androgenetic alopecia (androgen-dependent hair loss) [55] and atopic dermatitis characterized by chronic skin inflammation [56]. Interestingly, it has been proven that pretreatment of MSCs with bioactive compounds may enhance biological effects of MSC secretome. Yu et al. [57] pretreated human BMSCs (hBMSCs) with atorvastatin, which belongs to a statin class of medicines. Statins decrease the blood levels of low-density lipoproteins (LDLs) and are used in management of cardiovascular diseases. It has been demonstrated that exosomes isolated from hBMSCs pretreated with atorvastatin promote the proliferation, migration, and tube formation in human umbilical vein endothelial cells (HUVEC) and improve wound healing in rats with streptozotocin-induced diabetes by promoting blood vessels formation more potently than exosomes from nonpretreated hBMSCs [57]. On the clinicaltrials.gov website, there are already registered several clinical trials which investigate the usefulness of MSC secretome in treating the osteoarthritis and even investigating EVs in COVID-19, ischemic stroke, multiple system atrophy, and asthma [13].

## 5. Secretome in Nerve Injury Treatment

Peripheral nerve regeneration is a complex process that requires the involvement of many components, including ECM, cellular components, and neurotrophic factors, such as neurotrophins, e.g., BDNF or nerve growth factor (NGF) [58], expression of which have been described previously in ADSCs and BMSCs [59]. However, before the nerve regeneration begins after injury, the immune response occurs. Schwann cells are responsible for neuroinflammation, releasing proinflammatory cytokines and chemokines [9]. It is necessary to recruit macrophages, which remove degenerated axons and myelin debris. Nonetheless, inflammation should be inhibited so that regeneration can occur, and this process potentially may be accelerated by using MSC secretome. Immunosuppressive and anti-inflammatory properties of mesenchymal stromal cells growing *in vitro* are well documented [60]. MSCs exert their effects due to the secretion of interferon- (IFN-) *γ*, TGF-*β*, HGF, heme oxygenase-1, IL-6, and PGE2 [61]. Moreover, it has been proven that MSC exosomes can act as an immunomodulatory factors. It has been demonstrated that MSC-EVs increase M2 and decrease M1 macrophage infiltration and reduce the levels of IL-1*β* and tumor necrosis factor- (TNF-) *α* in the rat osteochondral defect model [62]. Similar results were reported by Sun et al. [63], as they showed that exosomes obtained from human umbilical cord-derived MSCs (UC-MSCs) switch macrophage polarization from M1 to M2 phenotype and improve mice functional recovery after spinal cord injury through downregulation of TNF-*α*, macrophage inflammatory protein-1*α* (MIP-1*α*), IL-6, and IFN-*γ*.

However, MSC secretome can exert beneficial effect not only in the terminal phase of inflammation but also in the regeneration process. Results of studies in animal models indicate that MSC exosomes are able to promote angiogenesis [64, 65]. Moreover, it has been shown *in vitro* and *in vivo* that human MSCs are capable of secreting neuroregulatory and neuroprotective factors, i.e., BDNF, ciliary neurotrophic factor (CNTF), glial cell line-derived neurotrophic factor (GDNF), or NGF thus increasing neuron viability; these are factors that increase neurogenesis or stimulate neurons proliferation and differentiation: bone morphogenetic proteins (BMP) family, granulocyte colony-stimulating factor (G-CSF), stem cell factor (SCF), TGF-*β*, and many others (e.g., IGF-1, HGF, vascular endothelial growth factor (VEGF), and stromal cell-derived factor- (SDF-) 1) involved in neurogenesis stimulation, apoptosis inhibition, glial scar formation, immunomodulation, and angiogenesis [66, 67]. Moreover, human MSC-conditioned media promotes survival of rat neurons and glial cells as well as show neuroprotective properties [68, 69]. There are also reports indicating that MSC exosomes promote axonal growth [70], inhibit neuronal cell apoptosis, suppress glial scar formation, and improve functional recovery after spinal cord injury in rats [71]. Moreover, secretome of human UC-MSCs pretreated with injured rat brain tissue extracts promotes neurogenesis by enhancing differentiation, maturation, and migration of newborn cells in the dentate gyrus of rats with traumatic brain injury and improve their cognitive functions significantly better than control UC-MSC secretome [72]. The properties of MSC secretome listed above and the possibility to modulate its biological effects by pretreatment of MSCs with bioactive compounds may be a significant factor justifying use of MSC secretome in peripheral nerve injury treatment. However, thus far, its positive effects have been proven in animal models and require direct confirmation for human ailments.

## 6. Conclusion

MSC secretome is a promising therapeutic tool for the treatment of facial nerve palsy. It combines the already proven therapeutic value of MSCs in regeneration of nerve fibers and treating nervous injuries with the product longevity, easy storage, and application expected from conventional pharmaceutical products. For these reasons, further investigation of MSC secretome potential is needed in order to produce (develop) the final product that will fulfill a need for a new therapy for facial nerve palsy.

## Data Availability

No underlying data was collected or produced in this study.
